# Autologous fibroblasts induce fibrosis of the nucleus pulposus to maintain the stability of degenerative intervertebral discs

**DOI:** 10.1038/s41413-019-0082-7

**Published:** 2020-02-13

**Authors:** Chen Chen, Tangjun Zhou, Xiaojiang Sun, Chen Han, Kai Zhang, Changqing Zhao, Xunlin Li, Haijun Tian, Xiao Yang, Yifan Zhou, Zhiqian Chen, An Qin, Jie Zhao

**Affiliations:** Shanghai Key Laboratory of Orthopaedic Implants, Department of Orthopaedic Surgery, Shanghai Ninth People’s Hospital, Shanghai Jiao Tong University School of Medicine, Shanghai, P. R. China

**Keywords:** Bone, Bone quality and biomechanics

## Abstract

Lumbar degenerative disc diseases cause low back pain (LBP). The maintenance of the height and stability of the intervertebral disc (IVD) space is an effective treatment for LBP. The following study evaluated the effects of fibroblast injection on intervertebral disc degeneration (IDD) in a preclinical setting. Compared with the IDD group, the fibroblast treatment group demonstrated effective maintenance of IVD height, reduced endplate degeneration, and improved nuclear magnetic resonance signals and overall histological structure. In doing so, fibrotic IVDs maintained the stability and biomechanics of the vertebra. This finding is in agreement with clinical findings that human nucleus pulposus (NP) fibrosis is essential for the maintenance of IVD height and mechanical properties in patients following percutaneous endoscopic lumbar discectomy (PELD). Mechanistically, we demonstrated that injected fibroblasts not only proliferated but also induced NP cells to adopt a fibrotic phenotype via the secretion of TGF-β. Finally, to better mimic human conditions, the efficacy of autologous fibroblast injection in the treatment of IDD was further examined in a nonhuman primate cynomolgus monkey model due to their capacity for upright posture. We showed that the injection of fibroblasts could maintain the IVD height and rescue IVD signals in cynomolgus monkeys. Taken together, the results of our study reveal that autologous fibroblast injection can enhance the natural process of fibrosis during acute and subacute stages of stress-induced IDD. Fibrotic IVDs can maintain the stability, biological activity, and mechanical properties of the intervertebral space, thus providing a new direction for the treatment of intervertebral space-derived lumbar degenerative diseases.

## Introduction

Low back pain (LBP) is a major cause of disability among elderly individuals and imposes substantial social and economic burden on societies worldwide.^1^ Lumbar/intervertebral disc degeneration (IDD) is one of the most common diagnoses involved in LBP and results in lumbar instability and height loss, which in turn leads to symptomatic nerve compression, intervertebral foramen stenosis, adjacent segment degeneration, and instability-induced LBP.^2^

The current treatment for IDD is based on a stepwise treatment paradigm largely limited to symptomatic relief using nonsteroidal or steroidal medication.^3^ Surgical interventions are often used for advanced-stage IDD^4^ to address severe neurological symptoms in an effort to restore the height and maintain stability of the intervertebral space.^5,6^ Percutaneous endoscopic lumbar discectomy (PELD) is one of the early surgical interventions employed to relieve nerve root decompression; however, the procedure is unable to restore the intervertebral space height or maintain the stability of the intervertebral space. As a result, patients often complain about LBP postoperatively with a high recurrence risk of neurological symptoms. To avoid these side effects, some end-stage surgical treatments including intervertebral disc fusion and fixation are commonly used. However, these procedures are not only ineffective in the long term but also exhibit inherent issues such as limited spinal motion and accelerated degeneration of adjacent intervertebral discs.^7^ Thus, more targeted and less invasive regenerative strategies are desperately needed, such as minimally invasive nucleus pulposus (NP) or annulus fibrosus (AF) treatments.

The IVD is a fibrocartilaginous tissue that sits between and connects two adjacent vertebral bodies and is composed of the external AF, the inner gel-like center NP, and the cartilaginous endplates that serve as a connection to the vertebrae.^8^ The external AF provides the mechanical and tensile strength of the disc to contain and maintain the osmotic pressure imposed by the NP. The AF encapsulates the gelatinous NP, which is composed predominantly of type II collagen and elastin fibers that encase proteoglycan aggregates, of which aggrecan is most abundant. The high levels of water molecules retained within the NP maintain the high hydrostatic swelling pressure that confers resistance to disc deformation and the maintenance of disc height. Cells are present at a low density within the abundant extracellular matrix (ECM) of the discs. As a result of restricted transport and low cellularity, disc repair is limited and particularly susceptible to injury and the aging-associated accumulation of tissue damage.

In the majority of human IDD cases, the degenerative process begins with the deterioration of NP tissue architecture. The NP begins to lose its ECM and its ability to maintain a significant hydrostatic pressure. Aggrecan content is reduced, and collagen composition changes from type II to type I, which can lead to fissures appearing in the NP and potentially extending into the AF.^9^ Gradual collagenization and calcification of the nucleus occurs, resulting in the degradation and reduction of ECM components in both the AF and NP. The consequences of these structural and architectural changes are decreased disc height and reduced ability of the spine to withstand compression.^10^ The biomechanical properties of the spine are compromised, leading to spinal instability, back pain and other clinical symptoms.

Due to the reasons described above, cell-based therapy may be a promising and effective approach for treating IDD. The choice of cell type is mainly focused on stem cells and NP cells. Clinically, we have found that fibrosis of the IVD after PELD can lead to stability of the spine. Thus, promoting fibrosis in IVD may offer a method for tissue repair and for helping stabilize the spine. Numerous promising studies have shown that dermal fibroblasts (DFbs) can integrate well into tissue defects and can accelerate fibrosis and tissue healing.^11^ In addition to their ease of accessibility, DFbs have gained much attention for use in cell therapy and tissue engineering. In general, fibroblasts conduct tissue repair through cell proliferation and formation of the extracellular matrix. Additionally, the growth factors that fibroblasts secrete and the fibroblast growth factors secreted by other cells can stimulate the proliferation of tissue-specific cells, enhancing the normal reparative process.^12,13^ Several independent studies have shown the potential for DFbs in the treatment of intervertebral disc degeneration and inflammation in rabbit models.^14,15^

Therefore, in this study, we explored the therapeutic effects of autologous DFb transplantation in IDD. In particular, we examined whether the transplantation of DFbs could reduce IVD degeneration and the early induction of reparative fibrosis to maintain the height of the IVD and to restore biomechanical function to the spine. We define reparative fibrosis as the formation of an organized fibrotic tissue that helps to mechanically stabilize the degenerating tissue defect. We believe that fibroblast-based cell therapy can be both clinically beneficial and commercially attractive for patients with the disease.

## Results

### Maintaining the stability of the degenerative IVD with fibroblast injection therapy

**Fig. 1:**
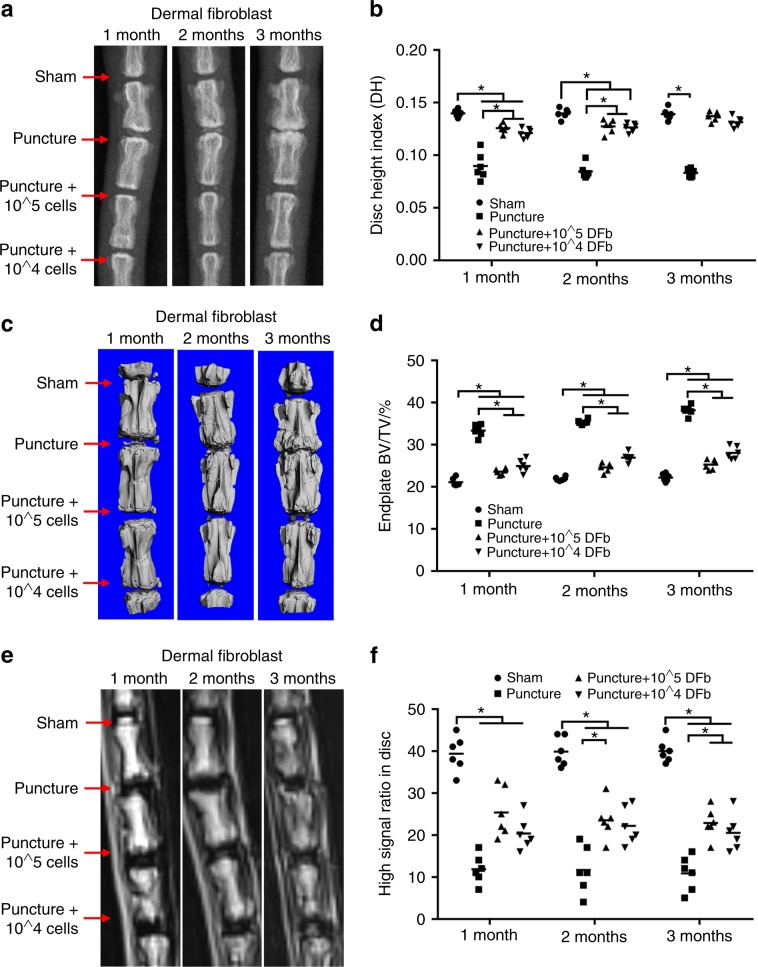
Injection of dermal fibroblasts (DFbs) into degenerative IVDs maintains disc height. The rat IDD model was established by caudal puncture of the IVD space using 20G needles without or with DFb injection treatment for 1, 2 or 3 months. **a**, **b** Radiographic images and disc height index (DHI), **c**, **d** 3D micro-CT reconstructions and endplate BV/TV measurements, and **e**, **f** MRI and disc degenerative degree (high signal ratio in disc) 1, 2, or 3 months after surgery and treatment were acquired and measured. Error bars represent the S.D., and significant differences were assessed with Student’s *t*-test; *P < 0.05

Using a needle puncture IVD degeneration model in rats,^16^ we assessed the therapeutic effect of the autologous injection of DFbs into the NP 1, 2, and 3 months postsurgery. Additionally, we evaluated the shorter term effects of DFb injection at 2 and 4 weeks after puncture surgery. All animals underwent X-ray, micro-CT and MRI scans to evaluate the effects of DFb therapy. As shown in Fig. 1a, b, X-ray scans of the IVD that underwent needle puncture showed time-dependent progressive loss of IVD height with increasing numbers of osteophytes in and around the intervertebral space. This culminated in segment fusion 3 months after surgery. On the other hand, for IVDs that received needle puncture and DFb injections (10^4^ or 10^5^ cells), disc heights were maintained with significantly less osteophyte formation around the IVD space over the 3-month period (Fig. 1a, b). Although a significant difference was observed 1 month postsurgery between the sham IVDs and DFb-treated IVDs, no difference was observed at 3 months. The IVDs that received needle puncture only showed progressive degeneration compared with sham and DFb-treated IVDs (Fig. 1b).

Micro-CT 3D reconstruction further confirmed the maintenance of IVD height and the reduction in osteophyte formation around the intervertebral space in DFb-treated IVDs (Fig. 1c). Micro-CT analysis of the lower end-plate of the upper vertebral body and the upper end-plate of the lower vertebral body showed significant increases in bone volume (BV/TV) in IVDs that received needle puncture only. Although the bone volume of the endplates in DFb-treated IVDs was higher than that in sham IVDs, the bone volume was significantly lower than that in puncture-only IVDs (Fig. 1d).

MRI scans further showed progressive degeneration of the puncture-only IVDs with the lowest gray levels 3 months postsurgery, and these IVDs exhibited the worst degenerative degree^17,18^ (Fig. 1e, f). No significant degeneration of the IVDs was observed in the sham group. The magnetic resonance T2 images revealed that IVDs were rescued to a certain extent following DFb therapy, with degenerative scores improving over time (Fig. 1e, f). Significant improvement was observed in degenerative status in DFb-treated IVDs at 3 months compared to that in puncture-only IVDs (Fig. 1f). The murine fibroblastic cell line L929 was used as a validation control for this procedure, and similar results were obtained (Supplementary Fig. 1a–f). We also confirmed similar improvements in disc height and degenerative scores at shorter time frames of 2 and 4 weeks after puncture surgery. As shown in Supplementary Fig. 2a–c, significant degeneration was observed 2 weeks after puncture surgery. DFb treatment 2 weeks after puncture surgery for 2 and 4 weeks (4 and 6 weeks, respectively) significantly improved the IVD height and degenerative degree compared with the puncture-only group (Supplementary Fig. 2a–c).

Collectively, the results from our in vivo rat IVD degenerative model showed that the injection of DFbs or the L929 murine fibroblastic cell line into the IVDs following needle puncture could maintain IVD height, reduce osteophyte formation in the adjacent vertebral bodies, inhibit adjacent end-plate degeneration, and improve the nuclear magnetic resonance signals of the IVDs.

### Autologous DFbs promote fibrosis of degenerative IVDs and maintain biomechanical properties

**Fig. 2:**
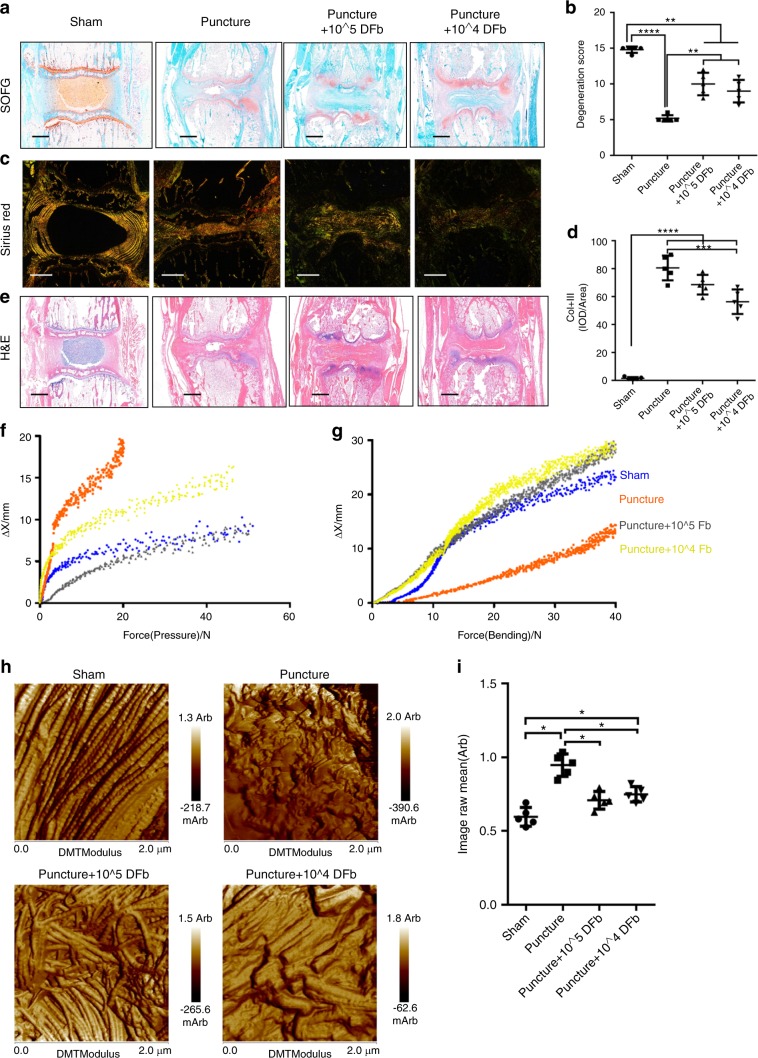
Autologous DFb injection promotes fibrosis of punctured IVDs and maintains the biomechanical properties of the discs. Histological examination and scoring of the IVDs under each experimental condition. **a**, **b** SOFG staining showing cartilage and proteoglycan distributions and degenerative scoring based on SOFG and H&E sections. **c** Sirius red staining under polarized light showing type I, II and III collagen distribution and orientation and the degree of fibrosis. **d** Integrated optical density (IOD) scoring of type I collagen (red and yellow) and type III collagen (green) based on Sirius red stained sections under each experimental condition. **e** H&E staining of tissue sections showing general cellular distribution and degree of degeneration in each experimental condition. **f** Pressure force was measured using a pressure clamp, and the pressure bearing/ΔX (position) fitting curve for each IVD was plotted. **g** The bending strength of IVDs was assessed on a bending fixture apparatus, and the bending force/ΔX (position) fitting curve for each IVD was plotted, where ΔX is the distance from the starting point to the measurement point. **h** Surface morphology of the IVDs under each experimental condition was visualized by tissue section atomic force microscopy (AFM). **i** The average Young’s modulus (modulus of elasticity; image raw mean) of IVDs under each experimental condition was quantified. Error bars represent the S.D., and significant differences were assessed with Student’s *t*-test; *P < 0.05, **P *<* 0.01, ***P < 0.001, and ****P < 0.000 1

Histological examinations of the IVDs were then carried out to assess the phenotypic changes following autologous DFb injection. In sham IVDs, SOFG (Fig. 2a), H&E (Fig. 2e) and Sirius Red (Fig. 2c) staining clearly demarcated NP-AF boundaries. SOFG staining revealed that the NP exhibited abundant proteoglycan content, and no significant disc degeneration was observed (Fig. 2a, b). On the other hand, NP-AF boundaries were not discernible in the experimental IVDs, and structures within the IVD space appeared disordered (Fig. 2a, c). The NP in the puncture-only IVD group was not centered, with fissures extending into the AF. In the DFb-treated IVDs, large amounts of regular fibrous tissues were visible with no obvious NP tissue structure or fissures. The extent of fibrosis formation increased with the increase in the number of DFbs injected. Sirius red staining performed under polarized light showed significant type I collagen arranged in a concentric lamellar pattern in the AF and type II collagen in the NP (unstained black region) in the sham IVD group (Fig. 2c). In the puncture-only IVD group, collagen orientation within the AF was disrupted, whereas in the DFb-treated IVD groups, abundant type I and type II collagen fibers were observed. Histological scoring of IDD showed that DFb-treated IVDs were significantly superior to those in the puncture-only group^19^ (Fig. 2b, d). Again, similar histological effects were observed in the shorter time frames of 2 and 4 weeks after puncture surgery. The IVDs in the puncture-only group exhibited significant degeneration compared to those in the sham controls 2, 4, and 6 weeks postsurgery. On the other hand, DFb treatment 2 weeks after puncture surgery for another 2 and 4 weeks (4- and 6-week time points, respectively) resulted in progressive fibrosis (Supplementary Fig. 3a, b).

Having established that autologous DFb treatment can maintain IVD height and reduce degenerative status, we then sought to examine the biomechanical properties of the IVDs.^20^ At the end of the 3-month experimental period, the rats were sacrificed, the tail spine was harvested, and biomechanical testing was performed. The IVDs corresponding to the sham or experimental conditions were divided from the center of the upper and lower adjacent vertebral bodies. Considering that IVD cell therapy was targeted at the intermediate stage of IDD, unfused segments with decreased IVD heights instead of spontaneously fused rat tail segments were selected for the puncture-only IVD group. Biomechanical analysis under pressure was performed using a pressure clamp, and the results showed a significant loss of IVD height in the puncture-only IVD group under low pressure (Fig. 2f), indicating a lack of biological effects of bearing pressure. For IVDs injected with DFbs, the IVD height improved when the disc was under pressure, with biomechanical effects of bearing pressure and pressure curves similar to those observed for sham IVDs (Fig. 2f). Next, we assessed the bending strength of the IVD segments using a bending fixture apparatus. As shown in Fig. 2g, the puncture-only IVDs exhibited significantly less bending properties under the same force. The bending properties of the DFb-injected IVDs were similar to those of the sham controls. These biomechanical assessments provided evidence that DFb-treated IVDs performed mechanically similar to normal IVDs in terms of bending strength and pressure loading/bearing.

Atomic force microscopy was then employed to examine the structural properties of the IVDs. As shown in Fig. 2h, sham IVDs demonstrated regularly distributed collagen fibers, whereas the collagen fibers in puncture-only IVDs were disorganized and disordered. Hyperplasia of distorted collagen fiber structures was visible in the DFb-treated IVDs (Fig. 2h). The average Young’s modulus of the sham IVDs was calculated to be the lowest at 0.596 8 ± 0.063 5 Arb and was within the range of 2.0 μm × 2.0 μm. The average Young’s modulus was highest in the puncture-only IVDs at 0.947 4 ± 0.075 9 Arb. The average Young’s modulus for the IVDs injected with 10^4^ and 10^5^ DFbs was 0.709 2 ± 0.059 8 Arb and 0.749 0 ± 0.052 0 Arb, respectively (Fig. 2i). The diagonals of four images were used to compare Young’s modulus at each position on the diagonals. The results showed that the puncture-only IVDs exhibited a significantly higher Young’s modulus than the sham controls.

The findings described above demonstrated that the injection of autologous DFbs into IVDs could induce fibrosis in degenerative IVDs, which helps to maintain IVD height. The biomechanical testing results further showed that the autologous injection of DFbs into IVDs could maintain the pressure-bearing and bending properties of the IVDs comparable to those of normal functioning IVDs.

### Fibrotic phenotype of intervertebral discs after discectomy

**Fig. 3:**
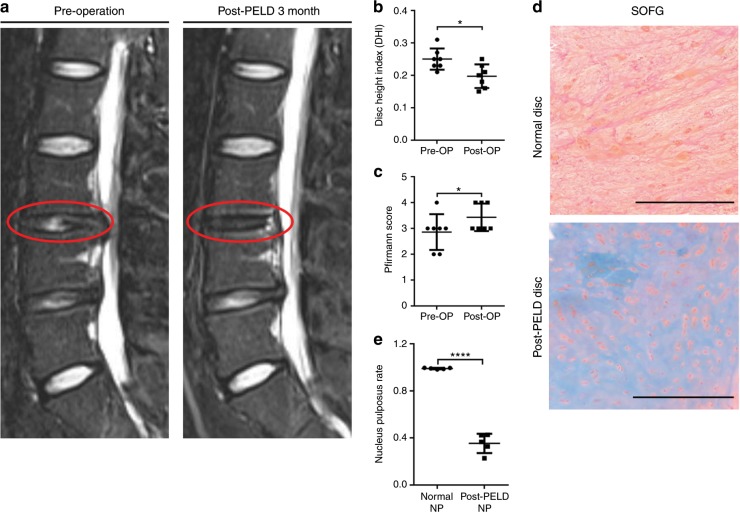
IVD fibrosis after percutaneous endoscopic lumbar discectomy (PELD). **a** T2-weighted STIR MRI of IVDs before and after PELD in patients with L3/4 disc herniation; L3, third lumbar vertebra; L4, fourth lumbar vertebra. **b**, **c** Disc height index (DHI) and degeneration Pfirrmann score for preoperative and post-PELD patients. Values represent the mean of seven IVDs from seven patients. **d** Representative SOFG-stained IVD images from normal and post-PELD patients. **e** Nucleus pulposus ratio (orange positive staining ratio) measured from SOFG-stained IVD images. Scale bars, 500 μm. Error bars the represent S.D., and significant differences were assessed with Student’s *t*-test, *P < 0.05, and ****P < 0.000 1

Figure 3a shows MRI scans of patients suffering from lumbar disc herniation both preoperation and 3 months after undergoing PELD. Compared to the preoperation conditions, post-PELD, a decrease in IVD height was observed (Fig. 3a, b). The whole IVD segment showed homogeneous intermediate and low signals on the MRI T2 STIR (Fig. 3a) and higher degenerative scores (Fig. 3c).

Histological assessment of normal disc tissues and post-PELD disc tissues was then carried out by staining with SOFG (Fig. 3d). In the normal tissues, a large number of red-stained chondrocytes and cell stroma were observed in the NP, while a significant reduction was found in patients who underwent PELD. Furthermore, the NP of patients who underwent PELD also exhibited large amounts of grayish-blue fibrous tissues and collagen fibers indicative of additional fibrotic characteristics (Fig. 3d). The NP to fibrosis ratio in post-PELD tissues was calculated to be 0.354 0 ± 0.036 8, further confirming that the IVDs from patients who underwent PELD were more fibrotic than normal IVDs (Fig. 3e).

### Fibroblasts can induce NP cells towards a fibrotic phenotype by activating TGF-β/Smad signaling

To examine the cellular effects of injected autologous DFbs on NP cells, we conducted in vitro cocultures of L929 fibroblastic cells and rat NP cells. Cocultures of human embryonic kidney HEK293T (293T) cells with rat NP cells were used as a negative control. The cell viability of both cell populations was not affected under coculture conditions. To induce fibroblastic cells into an inflammatory state, cocultures were stimulated with 1 μg·mL^−1^ LPS for 24 h. At the end of the experiment, RNA was extracted from the cells, and RT-qPCR analysis of gene expression was carried out using specific primers against rat fibroblast-specific protein-1 (FSP1), keratin-19 (KRT19), aggrecan, and type I and type II collagen. Previous studies have shown that KRT19 is one of the most suitable biomarkers for healthy NP cells, whereas aged or degenerative NP cells show reduced expression of KRT19.^21^ However, FSP1 is highly expressed in fibroblasts and is often used as a fibrotic biomarker.^22,23^ As shown in Supplementary Fig. 4a–e, the expression levels of FSP1 and type I collagen were significantly higher in cocultures of NP and L929 cells than in cocultures of NP and 293T cells. LPS stimulation significantly reduced the expression of FSP1 and type I collagen, particularly in NP and LP929 cocultures. On the other hand, the expression levels of the NP markers KRT19, aggrecan and type II collagen were markedly higher in NP and 293T cocultures than in NP and L929 cocultures before LPS stimulation. Again, LPS stimulation significantly reduced the expression of these NP markers, particularly in the NP and 293T cocultures. LPS stimulation did not markedly affect the expression of KRT19, aggrecan or type II collagen in NP and L929 cocultures (Supplementary Fig. 4a–e).

**Fig. 4:**
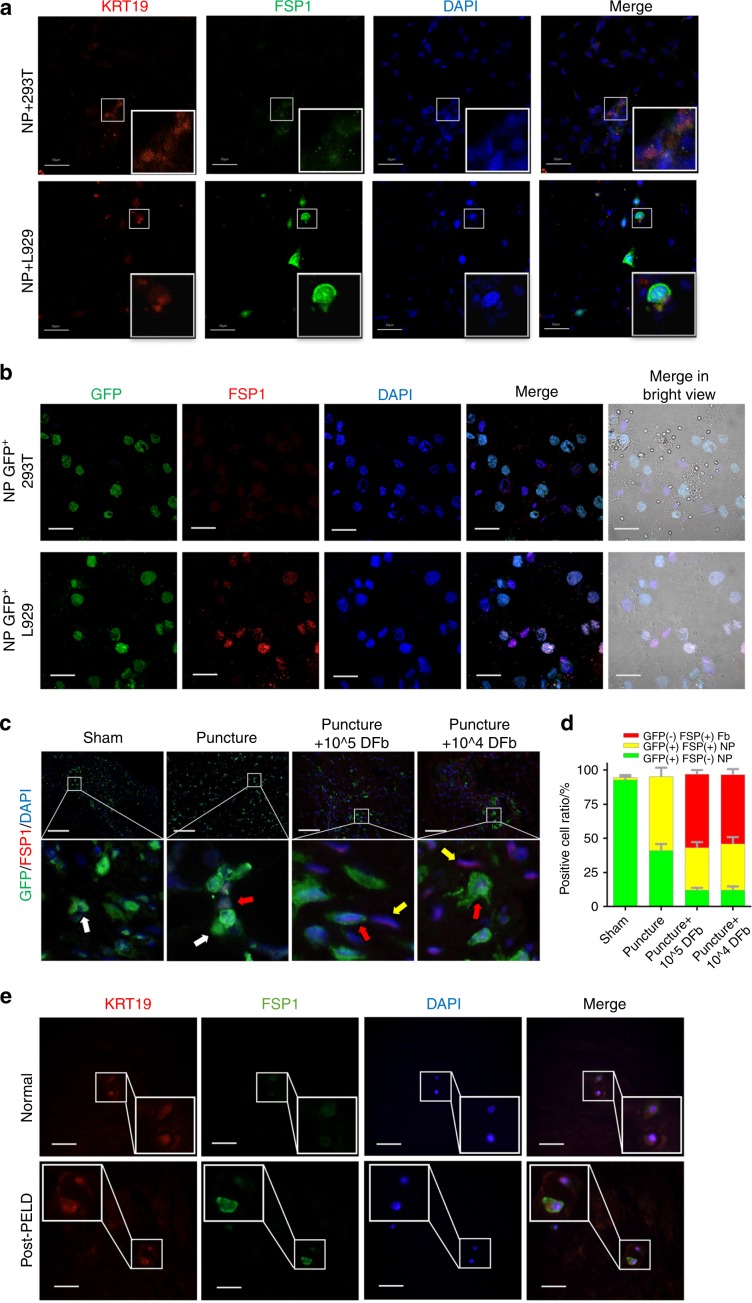
Fibroblasts induce NP cells towards a fibrotic phenotype. **a** Immunofluorescence analysis of KRT19 (red) and FSP1 (green) expression in the coculture system of NP cells with L929 or 293T cells. Nuclei were counterstained with DAPI (blue). **b** Immunofluorescence analysis of GFP (green) and FSP1 (red) expression in the coculture system of GFP^+ve^ NP cells with GFP^−ve^ L929 or 293T cells. Nuclei were counterstained with DAPI (blue). **c** Immunofluorescence images of the expression of FSP1 (red) in IVD tissue derived from GFP (green) transgenic rats under each experimental condition. Nuclei were counterstained with DAPI (blue). The white arrow shows GFP^+ve^ native and healthy NP cells. The red arrow indicates GFP^+ve^ and FSP1^+ve^ NP cells exhibiting a fibrotic phenotype. The yellow arrow indicates GFP^−ve^ and FSP1^+ve^ allogenic DFbs. **d** The proportions of GFP^−ve^ and FSP1^+ve^, GFP^+ve^ and FSP1^+ve^, and GFP^+ve^ and FSP1^−ve^ cells were quantified and plotted. **e** Immunofluorescence analysis of the expression of KRT19 (red) and FSP1 (green) in IVD tissue from normal and post-PELD patients. Scale bars, 10 μm. Error bars represent the S.D.

We further examined the protein expression of FSP1 and KRT19 using immunofluorescence microscopy. Under no LPS stimulation, we found high KRT19 but no FSP1 expression in NP and 293T cocultures (Fig. 4a, upper panels) and high levels of aggrecan and low levels of type I collagen (Supplementary Fig. 5a, upper panels), consistent with the NP phenotype. On the other hand, when NP cells were cocultured with L929 fibroblasts, both KRT19 and FSP1 were found to be expressed in the same cell (Fig. 4a, lower panels). Furthermore, the expression of aggrecan was found to be decreased, whereas type I collagen was increased (Supplementary Fig. 5a, lower panels). These results suggest that fibroblastic cells may induce NP cells to adopt a fibroblastic phenotype.

To further clarify the fibrotic changes in NP cells, GFP^+ve^ NP cells were cocultured with GFP^−ve^ L929 or 293T cells, and then the expression of GFP and FSP1 was examined under immunofluorescence. GFP^+ve^ NP cells cocultured with L929 cells were found to express both GFP and FSP1 (Fig. 4b lower panels). GFP^+ve^ NP cells cocultured with 293T cells only expressed GFP and did not express FSP1 (Fig. 4b upper panels). These results further demonstrate that under coculture conditions, fibroblastic cells can induce NP cells to adopt a more fibroblastic phenotype.

We also used GFP transgenic rats to analyze and further confirm the cellular effect of fibroblast therapy on IDD (Fig. 4c). Allogenic fibroblasts from WT rats that were negative for GFP were injected into the IVDs of GFP transgenic rats that had undergone needle puncture surgery. IVD tissue sections were harvested and stained with anti-FSP1 and DAPI and then analyzed by immunofluorescence microscopy. Native fibroblastic cells expressing GFP as well as FSP1 staining can be distinguished from allogenic fibroblasts, which will only stain for FSP1. As shown in Fig. 4c, d, only autologous cells that were GFP^+ve^ were observed in the IVDs of sham mice. A small population of cells that were GFP^+ve^ and FSP1^+ve^ were found in the IVDs from the puncture-only group. We believe that this could be the result of the natural transition to the degenerative fibrotic phenotype exhibited by NP cells. In the IVD groups injected with allogenic GFP^−ve^ fibroblasts, we found a mixed population of autologous NP cells that were GFP^+ve^ and FSP1^−ve^ as well as GFP^+ve^ and FSP1^+ve^ NP cells that had differentiated towards a fibrotic phenotype. A large population of GFP^−ve^ and FSP1^+ve^ allogenic fibroblasts were also observed (Fig. 4c, d).

We also examined the expression of KRT19 and FSP1 in normal IVD and post-PELD IVD tissues obtained from patients. As demonstrated in Fig. 4e, IVDs from patients who had undergone PELD were found to express both KRT19 and FSP1, further indicating fibrotic changes in the NP. These data suggest that fibrosis within the NP is a common occurrence and may be attributed to the natural healing process following PELD.

Next, the conditioned media from NP and L929 cocultures were subjected to ELISA testing to identify factors that will help us understand the mechanism by which fibroblastic cells can induce NP cells to adopt a fibrotic phenotype. Interestingly, we found that the TGFβ levels were substantially elevated in the conditioned media from NP and L929 cocultures (Supplementary Fig. 5b). Consistent with this finding, we observed higher expression of TGFβ and p-Smad2 in NP and L929 cocultures, but no expression of either was observed in NP and 293T cocultures (Supplementary Fig. 5a). We further examined the protein expression and activation states of Smad2 and Smad3, which are downstream effectors of TGFβ signaling, in NP cells treated with L929 conditioned media. Western blot analysis showed that the expression of p-Smad2 and p-Samd3 was elevated following treatment with L929 conditioned media, indicative of TGFβ signaling activation (Supplementary Fig. 5c). We verified this activation with the selective TGFβ receptor I kinase inhibitor LY364947, which successfully inhibited the phosphorylation of Smad2 and Smad3. Furthermore, we also found that the expression of TGFβ was significantly higher in IVDs injected with autologous DFbs than in IVDs from puncture-only and sham mice (Supplementary Fig. 5d, e).

**Fig. 5:**
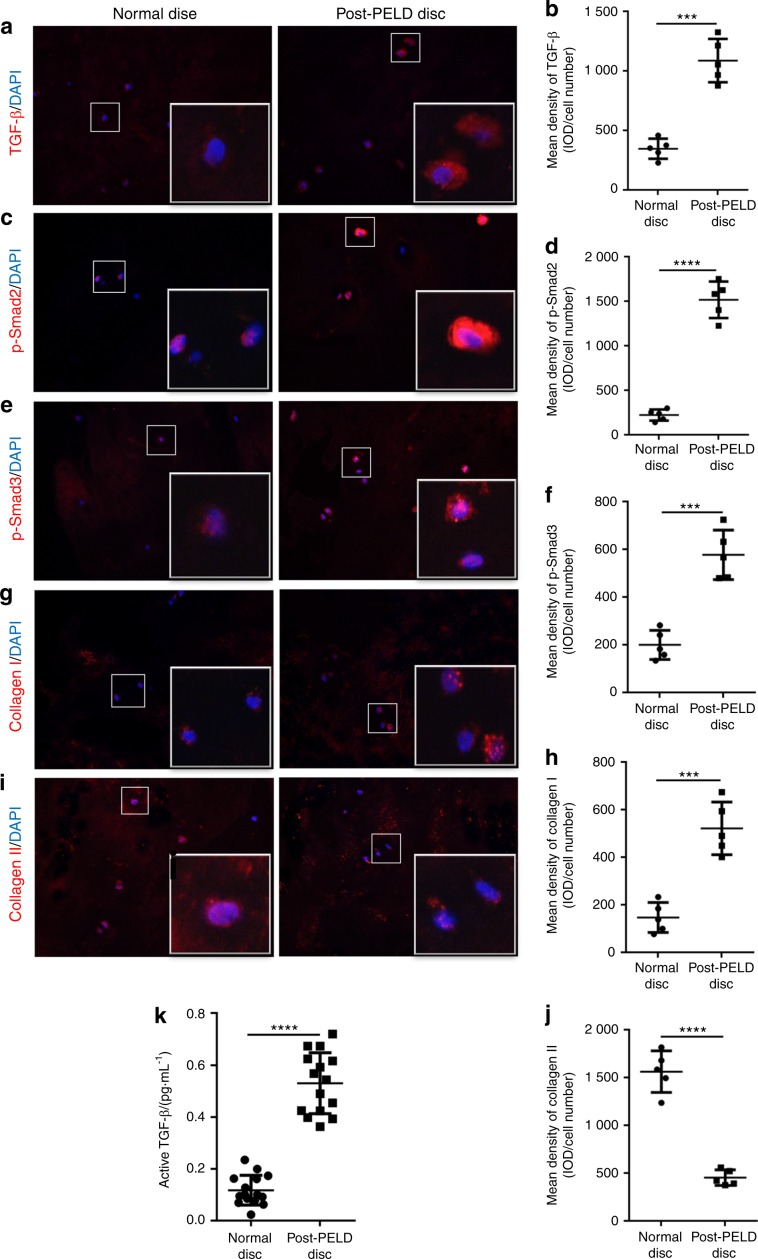
Expression of TGF-β and Smad2/3 in human fibrotic PELD IVDs. Immunofluorescence and quantitative analysis of the expression of **a**, **b** TGF-β, **c**, **d** phosphorylated Smad2, **e**, **f** phosphorylated Smad3, **g**, **h** type I collagen, and **i**, **j** type II collagen in IVD tissue from normal and post-PELD patients. **k** The concentration of serum TGF-β in normal patients and post-PELD patients was measured by ELISA. Values represent the mean of five discs from five donors. Error bars represent the S.D., and significant differences were assessed with Student’s *t*-test; ***P < 0.001, and ****P < 0.000 1

Previous studies have suggested a role for the TGFβ/Smad signaling pathway in maintaining the stability of the intervertebral space.^24^ Thus, to confirm that the TGFβ/Smad signaling pathway was upregulated in vivo, we again examined and compared normal IVD tissues with post-PELD IVD tissues from patients. Tissue sections were immunostained for TGFβ, p-Smad2, pSmad3, type I collagen and type II collagen and then assessed by immunofluorescence. As shown in Fig. 5a, b, TGFβ expression in the IVDs from post-PELD patients was significantly higher than that in the IVDs from normal healthy IVD patients. Consistent with elevated TGFβ expression, the levels of p-Smad2 (Fig. 5c, d) and p-Smad3 (Fig. 5e, f) in the post-PELD IVDs were also significantly higher than those in the normal healthy IVDs. In terms of collagen expression, type I collagen (Fig. 5g, h) was found to have much lower expression than type II collagen (Fig. 5i, j) in normal healthy IVDs. This is consistent with type II collagen being the structural component of the NP. On the other hand, type I collagen expression was markedly elevated in post-PELD IVDs (Fig. 5g, h), indicative of a fibrotic phenotype. Finally, blood serum samples from the patients further showed elevated concentrations of TGFβ in patients who underwent PELD compared with patients who did not (Fig. 5k).

Collectively, our data suggest that injected fibroblasts can induce NP cells to adopt a fibroblastic phenotype via the TGFβ/Smad signaling pathway, leading to reparative fibrosis of the IVD, which helps to suppress further degeneration of the intervertebral disc.

### Autologous fibroblast transplantation can decrease disc degeneration and maintain the intervertebral space in cynomolgus monkeys

**Fig. 6:**
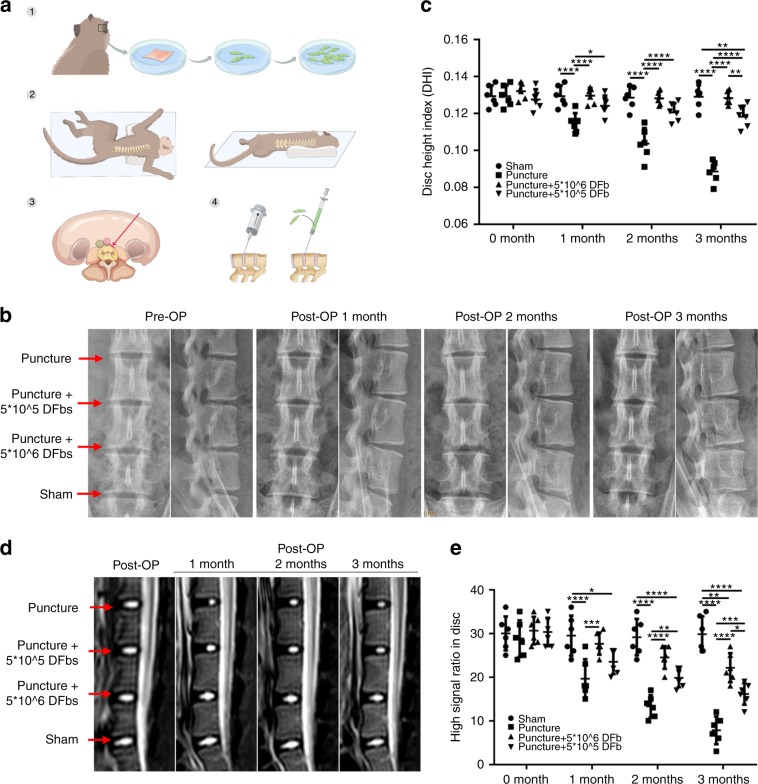
Autologous DFb therapy can restrain disc degeneration and maintain the intervertebral space in cynomolgus monkeys. **a** Experimental procedure of DFb extraction from cynomolgus monkeys and the OLIF surgical approach used to establish the IDD model and cell therapy approach. **b**, **c** Radiographic imaging and disc height index (DHI) measurements were conducted preoperatively and at 1, 2, and 3 months postoperatively. **d**, **e** MRI and quantitative measurements of the high signal area in the center of IVDs for each experimental condition were obtained preoperatively and 1, 2, and 3 months postoperatively. Error bars represent the S.D., and significant differences were assessed with Student’s *t*-test; *P < 0.05, **P < 0.01, ***P < 0.001, and ****P < 0.000 1

Having established the therapeutic benefits of fibroblast therapy in the small animal rat model, we wanted to see if we could induce the same effects in a large animal model with upright posture. Thus, we conducted fibroblast therapy using cynomolgus monkeys. Posterior auricular skin was collected for the culture of autologous DFbs from young cynomolgus monkeys aged between 6 and 7 years with a good IVD height and no significant IDD before the operation. The DFb culture was maintained to the third generation/passage, and then surgical treatment was performed. The lateral oblique lumbar interbody fusion (OLIF) surgical approach was used (Fig. 6a), with the L7/S1 IVD used as a sham control. L4/5, L5/6, and L6/7 were randomly divided into the puncture-only IVD, puncture + injection of 5 × 10^6^ DFb IVD, and puncture + injection of 5 × 10^5^ DFb IVD for intervention. After 2 months, the animals were subjected to X-ray and MRI. X-ray images showed significant loss of IVD height in the puncture-only IVDs (Fig. 6b, c), with MRI scans showing IDD and significant reduction in the hyperintense region in T2-weighted images (Fig. 6d). In comparison, IVDs injected with autologous DFbs showed maintenance of IVD height that was comparable to the maintenance observed in the presurgical procedure (Fig. 6b–d). Moreover, MRI scans further revealed that the hyperintense region in T2-weighted images increased significantly compared to that in the puncture-only group with improved IVD signals (Fig. 6d, e).

**Fig. 7:**
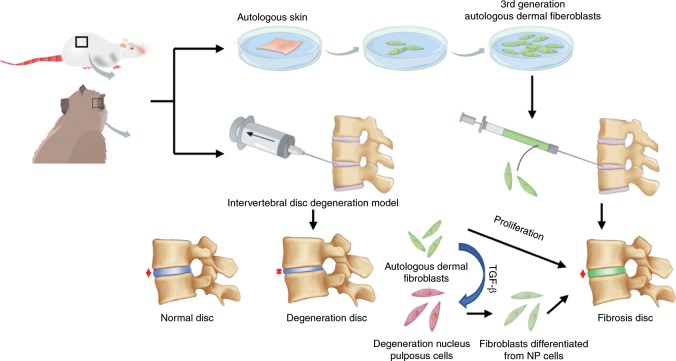
Schematic illustration of the potential underlying mechanism of fibroblast therapy. Autologous fibroblast injection induces NP cell trans-differentiation toward a fibroblastic phenotype, leading to extensive reparative fibrosis to maintain IVD height and restore the biomechanical properties of degenerative IVDs

Collectively, we have shown using both a small animal rat model and a large animal cynomolgus monkey model that fibroblast therapy can be used to maintain the IVD height and the biomechanical properties of degenerative IVDs. Mechanistically, fibroblast therapy halts further degenerative changes in the IVD by promoting reparative fibrosis and healing. Our data further suggest that autologous fibroblasts can induce native NP cells in degenerative IVDs to adopt a fibrotic phenotype via the activation of TGFβ/Smad signaling in order to enhance the reparative fibrosis and healing process (Fig. 7).

## Discussion

Currently, in the clinical setting, there is a lack of effective early and mid-term treatment options for lumbar intervertebral disc degeneration. At present, the end-stage surgical treatment for IDD is interbody fusion, which often limits lumbar motion and may cause other complications, such as accelerating the degeneration of adjacent lumbar segments. Thus, the development of a therapeutic approach that can alter the outcome of IDD and its associated morbidity will not only lead to significant cost savings for both patients and clinicians but also avoids unnecessary further surgical procedures and unwanted side effects.

Cell therapy is a promising approach to help manage IDD. In this study using the rat and monkey IDD models, we demonstrated that the autologous injection of DFbs into degenerative IVDs significantly increased disc height, inhibited adjacent end-plate degeneration, and improved the nuclear MRI signals of the treated IVDs. We believe this outcome was largely attributed to the induction of reparative fibrosis in the degenerative IVDs following the injection of autologous DFbs. In this study, we defined reparative fibrosis as the formation of organized fibrotic tissue that helped to mechanically stabilize the degenerating tissue defect. We propose that the induction of fibrosis by fibroblast therapy is a therapeutic treatment option for IDD. It has been well documented that extracellular matrix production and deposition as well as the release of TGFβ and other cell growth factors by fibroblasts are important for wound healing and tissue repair.^25,26^ The deposition of large amounts of extracellular matrix by fibroblasts during reparative fibrosis can serve to replenish what was lost during the degeneration of IVDs and can contribute to the maintenance of disc height. Although native healthy NP tissue has a higher ratio of proteoglycan to collagen and more water content than fibrotic tissue, which consists mainly of fibrocartilage, the ability of fibrosis alone to maintain the IVD height may alleviate pressure on sensitive tissues and restore the biomechanical function of IVDs, as we have shown with our biomechanical testing.^27^

Compared with other cell therapy approaches that utilize autologous NP or stem cell injection on the basis of restoring the number of functional NP cells to degenerative IVDs in order to initiate IVD regeneration,^28^ fibroblast therapy was found with our results to instead induce NP cells to adopt a fibrotic phenotype, possibly participate in reparative fibrosis and tissue healing processes via TGFβ/Smad signaling. High concentrations of active TGFβ have a definitive effect on induced matrix degradation,^29^ inflammation,^30,31^ cell migration and cartilage formation. A large number of studies have demonstrated that casein kinase inhibitors can reduce the fibrotic effect of TGFβ in pulmonary fibrosis.^32^ The TGFβ/Smad signaling pathway has been shown to play a key role in the fibrotic process.^24^ Numerous studies have shown that the dysregulation of TGF-β1/Smad represents an important pathogenic pathway in various tissue fibrogenesis, including lung, liver and kidney fibrosis.^33,34^ Of the Smads, Smad2 and Smad3 have been shown to be the major downstream regulators involved in TGF-β1-mediated tissue fibrosis.^35^ It has been shown that blockade of TGFβ/Smad signaling prevents or ameliorates tissue fibrosis;^36,37^ thus, the TGFβ/Smad signaling pathway has been a prime target for antifibrotic therapy. In this study, we found that autologous DFb treatment could induce NP cells to adopt a fibrotic phenotype via the TGFβ/Smad2/Smad3 pathway. Our data further indicate that the TGFβ/Smad pathway is important in the pathogenesis of IDD and the natural healing process following PELD. Despite studies investigating the antifibrotic potential of TGFβ/Smad inhibition, we believe that the early induction of TGFβ-mediated fibrosis by fibroblast injection intervention in the acute and subacute stages of IDD will benefit patients by stabilizing and maintaining disc height to minimize the loss of biomechanical functions of the intervertebral disc.

As IDD is extremely common, and with its substantial impact on disease burden in terms of lower back pain, numerous animal models have been developed in an effort to further understand and explore treatment options for this condition. However, many of these models were established using small animals, such as mice, rats and rabbits, which involve different characteristics from the human spine, particularly in terms of disc anatomy and mechanical loading of vertebrae. Human lumbar vertebrae are required to withstand pressure from more than 50% of the body weight that is further influenced by our upright posture, whereas the spine of quadrupeds, of which most models are based on, does not bear such vertical pressure in the resting state. As such, we conducted in vivo testing of our proposed fibroblast therapy in cynomolgus monkeys. Despite being quadrupedal for locomotion, cynomolgus monkeys also spend large amounts of time in semierect and erect positions and exhibit characteristics that more closely mimic humans, including IVD size, anatomy, spontaneous disc degeneration, and exposure to mechanical stresses compatible with erect posture. Consistent with our findings in the rat model, the results from the cynomolgus monkey model showed that treatment with autologous DFbs could minimize the loss of IVD height caused by degenerative changes with better MRI signal outcomes.

Indeed, the selection of patients who can benefit from this therapy is a very important consideration. In our opinion, the disease states that will benefit most from fibroblast injection intervention include acute disc herniation without neurological symptoms, acute disc herniation after simple NP removal and subacute stages of disc herniation without IVD collapse. The efficacy of autologous fibroblast therapy for chronic or recurrent disc herniation or for patients with severe loss of intervertebral disc height needs further investigation. In both models, we provided preliminary investigations of the effects of fibroblast injection therapy that mimic the acute or subacute stages of IDD. Although our results are promising with regard to the maintenance of disc height and biomechanical properties in early stages of disease, further long-term in vivo studies will be required to assess clinical translation, particularly in the aspects of reducing low back pain associated with chronic IDD. In addition, the long-term effects of autologous fibroblasts and fibrosis on native NP tissue structure and NP cell populations will also need to be evaluated. The underlying molecular mechanisms governing these changes and how NP cells can adopt a fibrotic phenotype require further investigation.

There are other limitations in this study that also need to be noted. First, the efficacy of fibroblast injection therapy on chronic or severe intervertebral degeneration with intervertebral disc collapse needs to be examined in both small and large upright animal models. In our monkey model of erect animals, we only examined the X-ray and MRI results; therefore, the histological and biomechanical analyses necessary for conclusive evidence of the beneficial effects of fibroblast therapy in upright animals were lacking. Detailed and comprehensive examinations of the effects of fibroblast therapy on potential neurological symptoms, including low back pain associated with IDD, are needed. Finally, the long-term effects of fibroblast therapy on surrounding tissues, such as cartilage and bone, will also need to be investigated.

In conclusion, despite some limitations, our pilot study provides evidence that fibrosis induced by autologous DFb injection into degenerative IVDs can conserve IVD height, which in turn maintains the mechanical properties of the IVD. Thus, fibroblast therapy may offer a potential therapeutic option for the treatment of IDD.

## Materials and methods

### Intervertebral disc specimens

Normal intervertebral disc tissues were collected from five patients: four males and one female. The average age of the patients was 49.7 ± 6.23 years (37–59 years). The enrolled subjects were patients who required discectomy and interbody fusion due to disc injury caused by trauma and fracture of a one-sided endplate extending into the intervertebral discs.

Specimens of fibrotic intervertebral discs were collected from five patients, two males and three females, with a combined average age of 63.4 ± 3.74 years (52–74 years). These patients had previously (over 1 year ago) undergone transforaminal PELD. In our standard procedure for PELD, only the protruding part of the NP and part of the NP around the broken AF were removed, with most of the NP remaining intact in the intervertebral disc. Moreover, these patients required discectomy and interbody fusion of the segments due to unalleviated or recurrent symptoms. The histological specimens from this group were obtained from discectomy during revision surgery. Intervertebral discs were sampled, and ELISA and immunofluorescence analyses were performed. The institutional ethics review board of Shanghai Ninth People’s Hospital, Shanghai Jiao Tong University School of Medicine approved this study (Approval # 2016-179-T123). Written informed consent was obtained from all patients.

### Dermal fibroblast isolation

All animals were anaesthetized with ketamine (75 mg·kg^−1^) and xylazine (10 mg·kg^−1^), and their dorsal hair was carefully removed. Full-thickness skin specimens were sterilized in 70% alcohol and then removed using surgical scissors. Skin specimens were washed in PBS to remove residual blood and then immersed in dispase solution (Dispase II Roche 4942078001, 2 mg·mL^−1^) at room temperature for 6 h with gentle agitation. The dermis was then carefully separated from the full-thickness skin specimens and soaked in collagenase solution (collagenase C7657 Sigma, 2 mg·mL^−1^) at 37 °C for 1 h with gentle agitation. The dermis/collagenase solution was then filtered through a 40 μm cell mesh, and the filtrate containing cells was centrifuged at 1 000 r·min^−1^ for 4 mins.^38^ Cell pellets were gently resuspended in fresh culture medium and incubated at 37 °C in a humidified atmosphere containing 5% CO_2_ and 95% air. Cells at passage 3 were used for downstream experiments.

### Small animal rat model

Six-week-old male Sprague-Dawley rats were obtained from Shanghai Lab, Animal Research Center Co. Ltd, Shanghai, China. Before proceeding with the incisions, all animals were anesthetized using an intraperitoneal injection of pentobarbital sodium (5 mg per 100 g of body weight), and their tail skin was sterilized with iodinated polyvinylpyrrolidone. A dorsal skin incision was then made to reveal the intervertebral disc. The tail vertebral discs, i.e., Co6/7 in the Sham group and Co7/8, Co8/9 and Co9/10 in the experimental groups, were punctured with a 20-gauge sterile needle from the dorsal to the ventral side of the tail. The needles were placed perpendicular to the skin to ensure that they were inserted at the center of the disc level through the AF into the NP.^16^ The needle head of the microinjector was limited to 5 mm in depth to ensure that the injection location was the center of the intervertebral disc. Subsequently, 1 × 10^5^ and 1 × 10^4^ rat DFbs and L929 cells were injected at Co8/9 and Co9/10, respectively. The skin was then sutured and disinfected once again. Mice were sacrificed at 4, 8, and 12 weeks postsurgery.

All animals were housed in an environment with a controlled temperature of 22 °C ± 1 °C, a relative humidity of 50% ± 1% and a light/dark cycle of 12/12 h. All animal studies (including the rat euthanasia procedure) were performed in compliance with the regulations and guidelines of Shanghai Jiao Tong University Institutional Animal Care and were conducted according to the AAALAC and IACUC guidelines.

### Large animal cynomolgus monkey model

Six young cynomolgus monkeys aged 6–7 years were obtained from Wincon TheraCells Biotechnologies Co. Ltd. (Nanning, China). The experimental and surgical procedures for the large animal studies were approved by the Animal Ethics Committee of the Shanghai Jiaotong University School of Medicine and were carried out in compliance with the regulations and guidelines of the Shanghai Jiao Tong University Institutional Animal Care and with the AAALAC and IACUC guidelines.

Monkeys were first placed into the right lateral recumbent position; the abdominal skin was then cut open, and the subcutaneous fat was separated layer by layer until the abdominal muscle layer was reached. The fascia of the obliquus externus abdominis was cut, and then the obliquus externus abdominis, obliquus internus abdominis and musculus transversus abdominis were bluntly separated. The transversalis fascia was bluntly passed through to enter the retroperitoneal space. The animals were then divided into the sham and experimental groups. Intervertebral disc L7/S1 was used in the sham group, while L4/5, L5/6 and L6/7 were used in the experimental groups. A negative-pressure puncture was performed using a 20-gauge needle with the depth of the needle head limited to 7 mm. Two groups were randomly selected from the three modeling segments, and then animals were injected with 5 × 10^6^ or 5 × 10^5^ autologous fibroblasts. After the operation, the skin was sutured layer by layer and disinfected.

### In vitro coculture of NP and fibroblastic cells

We used rat NP cells cocultured with mouse fibroblast L929 cells to imitate the *in vivo* cellular effects that fibroblasts may have on the NP cells within the intervertebral disc. NP cells were seeded in 6-well plates at a density of 1 × 10^6^ cells/well for 24 h. L929 or HEK293T cells (negative controls) at cell densities of 5 × 10^4^, 1 × 10^5^ and 2 × 10^5^ cells/well were then seeded into wells containing NP cells and were stimulated with or without 1 μg·mL^−1^ lipopolysaccharide (LPS) for 24 h. HEK293T cells were used as negative controls to account for the potential inhibitory effect on cell proliferation as a result of cell confluence due to the increased cell number of coculture conditions within the same well.

### RNA isolation and real-time quantitative PCR (RT-qPCR)

Total RNA was isolated from cells using a total RNA preparation kit (Axygen, NY, USA) in accordance with the manufacturer’s protocol. A first strand complementary DNA (cDNA) synthesis kit (TAKARA, Dalian, China) was used for the synthesis of cDNA from RNA templates. Relative mRNA expression was determined using the GoTaq 1-step real-time quantitative PCR (RT-qPCR) system (TAKARA) and qPCR using SYBR premix Ex Taq kit (TAKARA), and RT-qPCR was performed on an ABI Prism 7500 Fast Real-Time PCR System (Applied Biosystems, CA, USA). Primer sequences were designed using BLAST and are tabulated in Supplementary Table 1. The gene expression of GAPDH was used as an internal standard control. The expression levels of the target gene were obtained by calculating the ratio of cycle numbers of the initial exponential amplification phase as determined by the sequence detection system for specific target genes and GAPDH using the following formula: 2^−ΔΔCT^. The mean CT value of the target genes in the experimental group was normalized to the CT value of GAPDH to give a ΔCT value, which was further normalized to the control samples in order to obtain ΔΔCT. Three independent experiments were carried out, and all experiments were performed in triplicate.

### Protein extraction and western blot analyses

Serum-starved NP cells were treated with without 5 μmol·L^−1^ of the TGFβ R1 kinase inhibitor, LY364947, for 2 h and then stimulated with conditioned media from DFbs for 30 mins. Cells were then gently washed three times with PBS and lysed with RIPA buffer containing a protease inhibitor cocktail (Roche, Grenzach, Germany). Lysates were centrifuged at 9 168 *g* at 4 °C, and supernatants containing total cellular proteins were collected. Protein concentrations were quantified using a BCA protein quantification kit according to the manufacturer’s protocol (Thermo Fisher Scientific, Rockford, U.S.A.), and equal amounts of proteins (~30 μg) were resolved on 10%–12.5% SDS-PAGE gels. Separated proteins were electroblotted onto 0.22 μm PVDF membranes (Merck Millipore, CA, USA) overnight at 4 °C. Membranes were blocked with 5% skim milk in TBS-Tween (TBST) at room temperature (RT) for 1 h and then incubated with primary antibodies (1:1 000 dilution in 1% skim milk in TBST) at 4 °C overnight. The primary antibodies used included anti-phospho-Smad2, anti-phospho-Smad3, anti-Smad2/3 complex, and anti-GAPDH (all purchased from Cell Signaling Technology, Danvers, MA, U.S.A.). The next day, the membranes were washed three times with TBST and then incubated with anti-rabbit IgG conjugated with IRDye 800CW (LI-COR Biosciences, Lincoln, Nebraska, USA) for 1 h at RT. The immunoreactive bands were detected using the Odyssey Infrared Imaging System (LI-COR Biosciences). Positive immunoreactive bands were quantified with Image-Pro Plus 6.0 software (Media Cybernetics Inc., Rockville, MD, USA) and normalized to GAPDH.

### Histomorphometry and immunohistochemistry

NP tissues from patients or rats were fixed in 4% paraformaldehyde for 48 h and processed for histological sectioning. The tissue sections were stained with Safranin O-Fast Green (SOFG), Hematoxylin & Eosin (H&E) and Sirius Red. Histomorphometric analyses were based on a histological disc degeneration scoring system as described in Supplementary Table 2b.^39^ For immunohistochemistry, the tissue sections were sequentially placed in xylene I for 15 mins, xylene II for 15 mins, anhydrous ethanol I for 5 mins, anhydrous ethanol II for 5 mins, 85% alcohol for 5 mins, and 75% alcohol for 5 mins, and then were finally washed using distilled water. The tissue sections were placed in a repair kit (Servicebio, China) filled with EDTA antigen retrieval buffer (pH 9.0). The repair kit was then placed in a microwave oven for antigen retrieval for 10 mins at low heat. After natural cooling, the slides were placed in PBS (pH 7.4) and washed three times for 5 mins each. Sections were briefly air-dried, and then a PAP pen was used to demarcate boundaries around the tissues to prevent antibody overflow. An autofluorescence quencher was added to the tissue sections for 5 mins, rinsed with running water for 10 mins, and then blocked with BSA for 30 mins. The blocking solution was gently removed, and then the tissue sections were incubated with anti-KRT19 and anti-FSP1 antibodies (CST; 1:100) in a wet box at 4 °C overnight. The next day, the slides were immersed in PBS and washed 3 times for 5 mins each. The tissue sections were briefly air-dried and then incubated in the dark with cy3-goat anti-mouse (1:300; GB21301, Servicebio) and 488-goat anti-rabbit (1:400; GB25303, Sevicebio) for 50 mins at room temperature. The slides were again immersed in PBS, washed three times for 5 mins each, and then counterstained with DAPI solution for 10 mins at room temperature in the dark. The slides were subjected to final washes with PBS, air-dried and then sealed with anti-fluorescence quenching tablets. Tissue sections were observed under a fluorescence microscope, and digital images were acquired.

### Radiographic and MRI analysis

X-ray and MRI images of sedated SD rats were obtained 4, 8, and 12 weeks post-disc puncture and posttreatment. Digital X-ray images of the caudal vertebrae were recorded in the anteroposterior axis by a 21 lp/mm detector that provided up to 5X geometric magnification (Faxitron VersaVision; Faxitron Bioptics LLC, Tucson, AZ. USA). MRI was conducted on Siemens Magnetom Prisma E11 (Siemens Healthineers, Erlangen, Germany) with the following parameters: TR 3 000 ms, TE 80 ms, 1.1 mm thickness, 0.22 mm interval, FOV 160 mm × 65 mm, and voxel size 0.25 mm × 0.25 mm × 1.1 mm. Disc height indexes (DHIs) were calculated by three spinal surgeons who were blinded to the treatment groups as previously described with minor modifications (DHI = IVD height/adjacent IVD body height).^16^ The average IVD height was calculated by averaging the measurements obtained from the anterior, middle, and posterior portions of the IVD and dividing that average by the average of adjacent vertebral body heights. Intervertebral disc degeneration was also evaluated by three spinal surgeons using the Pfirrmann grading system (Supplementary Table 2a).^39^

### Micro-computed tomography analysis

At 1, 2, and 3 months postoperatively, SD rats were sacrificed, and spines were excised and fixed in a 1.5% phosphate-buffered glutaraldehyde solution. Micro-CT (SCANCO μCT 100; Brüttisellen, Zurich, Switzerland) with a spatial resolution of 30 mm was used to scan the specimen at a voltage of 70 kV and an electrical current of 200 mA. A region of interest was selected and reconstructed as well as analyzed as previously described^40^ using the associated analysis software. The bone volume/tissue volume (BV/TV) of the upper and lower end plates of each intervertebral disc were measured.

### Biomechanics and atomic force microscope

Biomechanical tests were carried out immediately after removal of the rat tail vertebral bodies, and the central intervertebral disc was completely preserved by cutting along the center of the vertebral body with a wire saw. Two vertebral specimens were embedded in polymethacrylic resin to preserve the structure and activity of the central intervertebral disc. The relationship between force and displacement was measured using ENGYI HY-0350 (HY-Instruments, Shanghai, China). A pressure clamp was used to measure the pressure of the intervertebral disc and the relationship between the pressure and the height of the intervertebral disc. The curvature of the intervertebral disc was examined with a bending fixture, and the relationship between the force of the curvature and the degree of motion of the intervertebral disc was measured.

The rat caudal vertebrae were frozen in the transverse position with the intervertebral disc as the center and stored at −20 °C. Atomic force microscopy (AFM) was conducted on a Bruker Dimension ICON (Bruker, Billerica, MA, USA) as previously described. For a wide range of undulating surfaces, the scanning rate of 0.7–2 Hz was used. A large scanning rate can reduce drift, but it is generally only used for scanning small flat surfaces. Measurements of disc surface were recorded to evaluate Young’s modulus and individual Young’s modulus.

### Statistical analysis

All experiments were conducted at least 3 times unless otherwise specified (Supplementary Table 3). Data from all experiments are presented as the mean ± S.D. The significant difference within each group was tested with Student’s *t*-test or one-way analysis of variance (ANOVA) using SPSS 19.0 software (IBM Corporation, New York, NY, USA). A significant difference was observed when the *P*-value was <0.05 or unless otherwise specified.

## Supplementary information

Supplementary Fig. 1

Supplementary Fig. 2

Supplementary Fig. 3

Supplementary Fig. 4

Supplementary Fig. 5

Supplementary Table 1

Supplementary Table 2

Supplementary Table 3

## Data Availability

The datasets used and/or analyzed during the current study are available from the corresponding author upon reasonable request.

## References

[1] Hill P (2001). Low back pain. N. Engl. J. Med..

[2] Meyer MA (2005). Persistent low back pain. N. Engl. J. Med..

[3] Wilkens P, Scheel IB, Grundnes O, Hellum C, Storheim K (2010). Effect of glucosamine on pain-related disability in patients with chronic low back pain and degenerative lumbar osteoarthritis: a randomized controlled trial. JAMA.

[4] Wang X, Wanyan P, Tian J, Hu L (2015). Meta-analysis of randomized trials comparing fusion surgery to non-surgical treatment for discogenic chronic low back pain. J. Back Musculoskelet. Rehabil..

[5] Panagiotacopulos ND, Knauss WG, Bloch R (1979). On the mechanical properties of human intervertebral disc material. Biorheology.

[6] Bydon M (2014). Lumbar fusion versus nonoperative management for treatment of discogenic low back pain: a systematic review and meta-analysis of randomized controlled trials. J. Spinal Disord. Tech..

[7] Sun Y (2012). Comparison of adjacent segment degeneration five years after single level cervical fusion and cervical arthroplasty: a retrospective controlled study. Chin. Med. J. (Engl.).

[8] Ziv I, Moskowitz RW, Kraise I, Adler JH, Maroudas A (1992). Physicochemical properties of the aging and diabetic sand rat intervertebral disc. J. Orthop. Res..

[9] Nerlich AG, Boos N, Wiest I, Aebi M (1998). Immunolocalization of major interstitial collagen types in human lumbar intervertebral discs of various ages. Virchows Arch..

[10] Teichtahl AJ (2015). A Dose-response relationship between severity of disc degeneration and intervertebral disc height in the lumbosacral spine. Arthritis Res. Ther..

[11] Frahs S (2018). Extracellular Matrix Expression and Production in Fibroblast-Collagen Gels: Towards an In Vitro Model for Ligament Wound Healing. Ann. Biomed. Eng..

[12] Specchia N, Pagnotta A, Toesca A, Greco F (2002). Cytokines and growth factors in the protruded intervertebral disc of the lumbar spine. Eur. Spine J..

[13] Xu X (2018). Transforming growth factor-β in stem cells and tissue homeostasis. Bone Res..

[14] Chee Ana (2016). Cell therapy with human dermal fibroblasts enhances intervertebral disk repair and decreases inflammation in the rabbit model. Glob. Spine J..

[15] Shi P (2019). Therapeutic effects of cell therapy with neonatal human dermal fibroblasts and rabbit dermal fibroblasts ondisc degeneration and inflammation. Spine J..

[16] Han B (2008). A simple disc degeneration model induced by percutaneous needle puncture in the rat tail. Spine.

[17] Walter BA (2017). MR elastography-derived stiffness: a biomarker for intervertebral disc degeneration. Radiology.

[18] Stolworthy DK (2015). MRI evaluation of spontaneous intervertebral disc degeneration in the alpaca cervical spine. J. Orthop. Res..

[19] Ohnishi T (2016). In Vivo Mouse Intervertebral Disc Degeneration Model Based on a New Histological Classification. PLoS One.

[20] Millecamps M, Czerminski JT, Mathieu AP, Stone LS (2015). Behavioral signs of axial low back pain and motor impairment correlate with the severity of intervertebral disc degeneration in a mouse model. Spine J..

[21] Lv F (2014). In search of nucleus pulposus-specific molecular markers. Rheumatol. (Oxf.).

[22] Strutz F (1995). Identification and characterization of a fibroblast marker: FSP1. J. Cell Biol..

[23] Sun L (2015). FSP1(+) fibroblast subpopulation is essential for the maintenance and regeneration of medullary thymic epithelial cells. Sci. Rep..

[24] Bian Q (2017). Mechanosignaling activation of TGFβ maintains intervertebral disc homeostasis. Bone Res..

[25] Stewart A, Thomas B, Koff J (2018). TGF-β: Master regulator of inflammation and fibrosis. Respirology.

[26] Perez-Cruet M (2018). Potential of human nucleus pulposus-like cells derived from umbilical cord to treat degenerative disc disease. Neurosurgery.

[27] Henry N, Clouet J, Le Bideau J, Le Visage C, Guicheux J (2018). Innovative strategies for intervertebral disc regenerative medicine: from cell therapies to multiscale delivery systems. Biotechnol. Adv..

[28] Xie Z (2018). TGF-β synergizes with ML264 to block IL-1β-induced matrix degradation mediated by Krüppel-like factor 5 in the nucleus pulposus. Biochim Biophys. Acta Mol. Basis Dis..

[29] Zhang J (2017). TGF-β1 suppresses CCL3/4 expression through the ERK signaling pathway and inhibits intervertebral disc degeneration and inflammation-related pain in a rat model. Exp. Mol. Med..

[30] Yang H (2015). TGF-βl suppresses inflammation in cell therapy for intervertebral disc degeneration. Sci. Rep..

[31] Wen X (2018). LOXL2, a copper-dependent monoamine oxidase, activates lung fibroblasts through the TGF-β/Smad pathway. Int J. Mol. Med..

[32] Yoshida K, Murata M, Yamaguchi T, Matsuzaki K (2014). TGF-beta/Smad signaling during hepatic fibro-carcinogenesis (review). Int J. Oncol..

[33] Chen L (2018). Central role of dysregulation of TGF-β/Smad in CKD progression and potential targets of its treatment. Biomed. Pharmacother..

[34] Eser PO, Janne PA (2018). TGFβ pathway inhibition in the treatment of non-small cell lung cancer. Pharmacol. Ther..

[35] Loboda A, Sobczak M, Jozkowicz A, Dulak J (2016). TGF-β1/Smads and miR-21 in renal fibrosis and inflammation. Mediators Inflamm..

[36] Higgins SP (2018). TGF-β1/p53 signaling in renal fibrogenesis. Cell signal..

[37] Yang L (2016). Taurine reduced epidural fibrosis in rat models after laminectomy via downregulating EGR1. Cell Physiol. Biochem.

[38] Rinkevich Y (2015). Skin fibrosis. Identification and isolation of a dermal lineage with intrinsic fibrogenic potential. Science.

[39] Boos N (2002). Classification of age-related changes in lumbar intervertebral discs: 2002 Volvo Award in basic science. Spine.

[40] Rutges J (2011). Micro-CT quantification of subchondral endplate changes in intervertebral disc degeneration. Osteoarthr. Cartil..

