# Supplementary material for: Autologous fibroblasts induce fibrosis of the nucleus pulposus to maintain the stability of degenerative intervertebral discs
Source: Bone Res. 2020 Feb 13;8:7. doi: 10.1038/s41413-019-0082-7 (PMC7015945; doi:10.1038/s41413-019-0082-7)
Supplement: Supplementary file 6 — Supplementary Table 1 [file 41413_2019_82_MOESM6_ESM.pdf]

Table 1 primers used in the qRT-PCR experiment

Gene	Accession Number	Description	5'-Primer-3'
Rat			
FSP1	NM_012618.2	Forward	AGCACTTCCTCTCTCTTGGT
Fibroblast Special Protent 1		Reverse	GTCCCTGTTGCTGTCCAAGT
KRT19	NM_199498.2	Forward	CCATGTCTTCCTATGGGGGC
keratin 19		Reverse	GAGGCGATCGTTCAGGTTCT
ACAN	NM_022190.1	Forward	CAAGTCCCTGACAGACACCC
Aggrecan		Reverse	GTCCACCCCTCCTCACATTG
Col1a1	NM_053304.1	Forward	GGATCGACCCTAACCAAGGC
Collagen type I alpha 1		Reverse	GATCGGAACCTTCGCTTCCA
Col2a1	NM_012929.1	Forward	GGCCAGGATGCCCGAAAATTA
Collagen type II alpha 1		Reverse	GGCTGCAAAGTTTCCTCCAC
GAPDH	NM_017008.4	Forward	AGTGCCAGCCTCGTCTCATA
glyceraldehyde-3-phosphate dehydrogenase		Reverse	GGGTTTCCCGTTGATGACCA
