# Supplementary material for: Autologous fibroblasts induce fibrosis of the nucleus pulposus to maintain the stability of degenerative intervertebral discs
Source: Bone Res. 2020 Feb 13;8:7. doi: 10.1038/s41413-019-0082-7 (PMC7015945; doi:10.1038/s41413-019-0082-7)
Supplement: Supplementary file 7 — Supplementary Table 2 [file 41413_2019_82_MOESM7_ESM.pdf]

Grading System

a. Pfirrmann Grading System (Evaluation of human intervertebral disc degeneration by MRI)

	Pfirrmann Grading System
Grade I	Homogeneous bright white structure
Grade II	Inhomogeneous white structure， possible horizontal bands
Grade III	Clear distinction between annulus and nucleus
Grade IV	No collapsed disc space
Grade V	Collapsed disc space

b. Han Grading System (Evaluation of rat intervertebral disc degeneration by HE staining)

I. Cellularity of the anulus fibrosus	Grade
Fibroblasts comprise more than 75% of the cells	1
Neither fibroblasts nor chondrocytes comprise more than 75% of the cells	2
Chondrocytes comprise more than 75% of the cells	3
II. Morphology of the anulus fibrosus	
Well-organized collagen lamellae without ruptured or serpentine fibers	1
Inward bulging, ruptured or serpentine fibers in less than one third of the annulus	2
Inward bulging, ruptured or serpentine fibers in more than one third of the annulus	3
III. Border between the anulus fibrosus and nucleus pulposusu	
Normal, without any interruption	1
Minimal interruption	2
Moderate or severe interruption	3
IV. Cellularity of the nucleus pulposus	
Normal cellularity with stellar shaped nuclear cells evenly distributed throughout the nucleus	1
Slight decrease in the no. of cells with some clustering	2
Moderate or severe decrease (50%) in the number of cells with all the remaining cells clustered and separated by dense areas of proteoglycans	3
V. Morphology of the nucleus pulposus	
Round, comprising at least half of the disc area in midsagittal sections	1
Rounded or irregularly shaped, comprising one quarter to half of the disc area in midsagittal sections	2
Irregularly shaped, comprising less than one quarter of the disc area in midsagittal sections	3
