# Supplementary material for: Autologous fibroblasts induce fibrosis of the nucleus pulposus to maintain the stability of degenerative intervertebral discs
Source: Bone Res. 2020 Feb 13;8:7. doi: 10.1038/s41413-019-0082-7 (PMC7015945; doi:10.1038/s41413-019-0082-7)
Supplement: Supplementary file 8 — Supplementary Table 3 [file 41413_2019_82_MOESM8_ESM.pdf]

	Figure1		
	X-ray	Micro-CT	MRI
1 month	6	6	6
2 month	6	6	6
3 month	6	6	6

	Figure2				
	SOFG	Sirius Red	H&E	biomedical testing	AFM
Sham	5	5	5	3	5
Puncture	5	5	5	3	5
Puncture+10^5DFb	5	5	5	3	5
Puncture+10^4DFb	5	5	5	3	5

	Figure3
MRI	7
Intervertebral disc specimens	5 patients normal disc<br>5 patients post-PELD disc

	Figure4		
	Co-culture	GFP+ Rat	Intervertebral disc specimens
	3	3 rats each group	5 patients normal disc<br>5 patients post-PELD disc
Repetitions	3	3	3

	Figure5	
	Immunofluorescence	Elisa
Normal disc	5 patients	5
Post-PELD disc	5 patients	5
Repetitions		3

	Figure6	
	X-ray	MRI
1 month	6	6
2 month	6	6
3 month	6	6

	Supplementary Figure1		
	X-ray	Micro-CT	MRI
1 month	6	6	6
2 month	6	6	6
3 month	6	6	6

	Supplementary Figure2	
	X-ray	MRI
1 month	6	6
2 month	6	6
3 month	6	6

	Supplementary Figure3		
SOFG/H&E	1 month	2 month	3 month
Sham	5	5	5
Puncture	5	5	5
Puncture+10^5DFb	5	5	5
Puncture+10^4DFb	5	5	5

	Supplementary Figure4	Supplementary Figure5			
	PCR	ELISA	WB	Co-culture	Immunofluorescence
Repetitions	3	7	3	3	5
